# Supplementary material for: SBMLtoOdin and Menelmacar: interactive visualisation of systems biology models for expert and non-expert audiences
Source: Bioinformatics. 2025 Sep 2;41(9):btaf484. doi: 10.1093/bioinformatics/btaf484 (PMC12472120; doi:10.1093/bioinformatics/btaf484)
Supplement: btaf484_Supplementary_Data [file btaf484_supplementary_data.pdf]

Tool	Translates SBML into	Input	Simulations	Can change parameters in simulations	Interactive visualisation
`SBMLtoOdin` and `Menelmacar`	odin	BioModels ID, any SBML model file	local, in browser	yes	yes
BioSimulations/ BioSimulators	/	Models in BioSimDB COMBINE/OMEX archive	on server	yes	no
JWS Online	/	Models in JWS database	on server	no	no
libRoadRunner 2.0	/	e.g. BioModels url, any SBML model file	local	yes	no
Systems Biology Format Converter (SBFC)	e.g. MATLAB, Octave, XPP	e.g. BioModels url, any SBML model file	no	no	no
SBMLtoODEpy	Python	any SBML model file	no	no	no

Table 1: Comparison of `SBMLtoOdin` and `Menelmacar` to other tools for translating and simulating SBML models.
